# Male reproductive phenotypes of genetically altered laboratory mice (*Mus musculus*): a review based on pertinent literature from the last three decades

**DOI:** 10.3389/fvets.2024.1272757

**Published:** 2024-03-04

**Authors:** Kakanang Buranaamnuay

**Affiliations:** Molecular Agricultural Biosciences Cluster, Institute of Molecular Biosciences (MB), Mahidol University, Nakhon Pathom, Thailand

**Keywords:** fertility, mouse, mutant, reproductive organs, sperm

## Abstract

Laboratory mice (*Mus musculus*) are preferred animals for biomedical research due to the close relationship with humans in several aspects. Therefore, mice with diverse genetic traits have been generated to mimic human characteristics of interest. Some genetically altered mouse strains, on purpose or by accident, have reproductive phenotypes and/or fertility deviating from wild-type mice. The distinct reproductive phenotypes of genetically altered male mice mentioned in this paper are grouped based on reproductive organs, beginning with the brain (i.e., the hypothalamus and anterior pituitary) that regulates sexual maturity and development, the testis where male gametes and sex steroid hormones are produced, the epididymis, the accessory sex glands, and the penis which involve in sperm maturation, storage, and ejaculation. Also, distinct characteristics of mature sperm from genetically altered mice are described here. This repository will hopefully be a valuable resource for both humans, in terms of future biomedical research, and mice, in the aspect of the establishment of optimal sperm preservation protocols for individual mouse strains.

## Graphical abstract

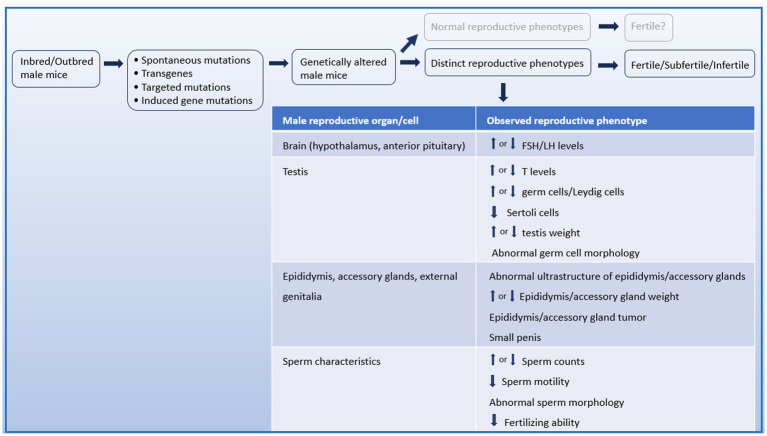

## Introduction

Laboratory mice, hereinafter refer to as mice, usually belong to the *Mus musculus* species. With their biological characteristics and the close similarity to humans in genetics, anatomy, and physiology, they are the preferred laboratory animals used for biomedical research (1). There are hundreds of established inbred, outbred, and genetically altered mouse strains. An inbred strain is a group in which all individuals are nearly genetically identical. In reference to mice, this is accomplished through brother–sister or offspring-parent matings for at least 20 consecutive generations (2). As a result, this type of mice is theoretically free of genetic variants and is suitable for studying the roles of particular genes or performing experiments that exclude genetic variation as a factor (3). On the contrary, outbred mice, produced by random mating systems and consequently having inter-individual genetic variation, are only used as research models when identical genotypes are dispensable or a population with genetic variation is required (4). Noticeably, outbred populations are generally called stocks rather than strains. Genetically altered mouse strains with genomic mutations have been produced by a number of procedures including ordinary breeding and inbreeding (random spontaneous mutations), transgenes (which foreign genetic materials are introduced into mice genome), targeted mutations (knock-out and knock-in), and virus-induced or chemically-induced gene mutations (5–7). Genetically altered mice are useful for elucidating fundamental biological processes, studying associations between gene mutations and disease phenotypes, and modeling human disease mechanisms (8). This paper compiling most existing relevant literature published in peer-reviewed journals during the last three decades and indexed in PubMed aims to describe the distinct, especially abnormal, male reproductive phenotypes of various genetically altered mouse strains. Such phenotypes covering from the brain through the male reproductive organs and germ cells were intentionally generated for research or naturally/accidentally found (Table 1). This repository will more or less enable researchers (1) to gain novel information or brush up on their knowledge, (2) to identify suitable mouse strains for their future studies, and (3) to identify potential research topics to explore next.

**Table 1:** Male reproductive phenotypes and fertility status of some genetically altered mice.

Mouse genotype	Method to generate genetically altered mice	Reproductive organs as the origin of disorders	Observed reproductive phenotypes				Fertility status	References
			Brain (hypothalamus and anterior pituitary gland)	Testis (internal cells, structure, and function)	Epididymis, accessory sex glands, and external genitalia	Sperm characteristics		
*Csfm^op^*/*Csfm^op^*	Targeted mutations (knockout)	Hypothalamus, testis (Leydig cells)	Low LH levels	Low T levels, abnormal Leydig cell ultrastructure		Low sperm counts and viability	Infertile	(9–13)
Gpr54	Targeted mutations (knockout)	Hypothalamus	Low FSH, LH levels	Low T levels, decreased germ cell and Sertoli cell numbers, small testis	Undeveloped accessory glands, small penis, reduced anogenital distance		Infertile	(14, 15)
Kiss1	Targeted mutations (knockout)	Hypothalamus	Low FSH, LH levels	Low T levels, decreased germ cell numbers, small testis	Undeveloped accessory glands, small penis, reduced anogenital distance		Infertile	(14, 15)
GNR23	Transgenes	Hypothalamus	Low FSH levels	Low testicular weight			Fertile	(16)
*Cdk4^−/−^*	Targeted mutations (knockout)	Anterior pituitary gland	Low FSH levels	Adult (>4 month-old): increased germ cell apoptosis, decreased germ cell numbers, atrophic testis	Atrophic epididymis	Low sperm counts	Infertile	(17, 18)
*FSHβ^−/−^*	Targeted mutations (knockout)	Anterior pituitary gland	No FSH	Decreased germ cell and Sertoli cell numbers, small testis			Fertile	(19, 20)
*ActRIIA^−/−^*	Targeted mutations (knockout)	Anterior pituitary gland, testis	Low FSH levels	Decreased germ cell and Sertoli cell numbers, small testis		Low sperm counts	Fertile	(19, 20)
*md^nc^*	Spontaneous mutations	Anterior pituitary gland	Low FSH, LH levels	Low E, T levels		Low sperm counts and motility	Infertile	(21)
*Hoxa-10^−/−^*	Targeted mutations (knockout)	Testis (gubernaculum)		Cryptorchid testis, small seminiferous tubule diameter, decreased germ cell numbers, and vacuolated Sertoli cells	Diminished depth and complexity of seminal vesicle folding, small coagulating gland		Infertile	(22–24)
ΔdblGATA	Transgenes	Testis (Sertoli cells, and Leydig cells), prostate gland	High LH levels	Low T levels, expanded interstitial compartments, increased Leydig cell numbers	Decreased accessory gland weight, reduced anogenital distance	Abnormal sperm morphology (amorphous heads)	N/A	(25)
*Nptn^−/−^*	Targeted mutations (homologous recombination)	Testis (germ cells, Sertoli cells, and Leydig cells)		Low T levels			Infertile (lack mounting behavior)	(26)
*Sdc1^+/−^*	Targeted mutations (knockout)	-		Low testicular weight	Low epididymal weight		Possibly fertile	(27)
*Atm* ^*−*/−^	Targeted mutations (knockout)	Testis (germ cells)		Degenerated germ cells, abnormal Sertoli cells, and atrophic testis			Infertile	(28, 29)
*Bcl-xL*	Transgenes	Testis (germ cells)		Young: premeiotic germ cell accumulation, enlarged testis			Infertile	(30)
				≥5 week-old: decreased germ cell numbers, testicular shrinkage, and multinucleated giant cell presence				
*Bcl-2*	Transgenes	Testis (germ cells)		Young: premeiotic germ cell accumulation, enlarged testis			Infertile	(30)
				≥5 week-old: decreased germ cell numbers, testicular shrinkage, and multinucleated giant cell presence				
*Bax^−/−^*	Targeted mutations (knockout)	Testis (germ cells)		Young: premeiotic germ cell accumulation, enlarged testis			Infertile	(31, 32)
				Adult: decreased germ cell numbers, testicular shrinkage				
ROSA41	Targeted mutations (gene trapping)	Testis (germ cells)	High FSH levels	Degenerated germ cells, no mature sperm, decreased Sertoli cell and Leydig cell numbers, and atrophic testis	Atrophic seminal vesicle		Infertile	(33)
*bcl-w^−/−^*	Targeted mutations (homologous recombination)	Testis (germ cells, Sertoli cells)		≥4 week-old: increased germ cell apoptosis, increased Leydig cell numbers, decreased germ cell and Sertoli cell numbers, and low testicular weight		No spermatozoa	Infertile	(34)
*bcl-w*	transgenes	Testis		≥1 week-old: decreased germ cell numbers, multinucleated giant cell presence, vacuolated seminiferous epithelium, and low testicular weight		Low sperm counts	Infertile	(35)
c-FLIP_L_	Transgenes	Testis (germ cells)		≥8 week-old: increased germ cell apoptosis, decreased germ cell numbers, multinucleated giant cell presence, atrophic seminiferous tubules, bilateral testicular shrinkage (≥8 month-old)		Low sperm counts and motility	Fertile	(36)
ERKO	Targeted mutations (knockout)	Testis (efferent ductules), head of epididymis		Young: accumulated testicular secretions, swollen seminiferous tubules, enlarged testis		Low sperm counts, motility, and fertilizing ability	Infertile	(37–39)
				Adult (≥5 month-old): atrophic testis				
*Lgr4^Gt/Gt^, Lgr4^−/−^*	Targeted mutations (gene trapping)	Testis (efferent ductules), head of epididymis		Accumulated spermatozoa, leukocytes, and testicular secretions, swollen rete testis and efferent ductules	Short, swollen, and/or ruptured epididymal head, accumulated leukocytes in epididymal lumen, low epididymal weight	Low sperm motility and viability, abnormal sperm morphology (cytoplasmic droplet, hairpin structure at the tail)	Infertile	(40, 41)
*VEGF*	Transgenes	Testis (Sertoli cells, Leydig cells), epididymis		Adult (≥6 month-old): vacuolated seminiferous epithelium, few/no germ cells, increased size and density of capillaries in the testis, swollen rete testis	Low columnar epithelial cells in the epididymal head, increased size and number of capillaries in the epididymis, swollen epididymis		Infertile	(42)
*CDC37/c-myc*	Transgenes	Testis (Leydig cells)		Adult (≥10 month-old): Leydig cell tumor, testicular hyperplasia			N/A	(43)
Cyclin D1	Transgenes	Testis		Adult (≥16 month-old): testicular sarcoma			N/A	(44)
*Cdk4* ^R24C/R24C^ *, Cdk4* ^+/R24C^	Targeted mutations (knock-in)	Multiple organs including reproductive system	Pituitary tumors (adenoma, carcinoma)	Leydig cell tumor	Seminal vesicle tumor (adenoma)		N/A	(45)
*Prl3c1^−/−^*	Targeted mutations (knockout)	Testis (Leydig cells)	High LH levels	High T levels, enlarged testis, expanded interstitial compartments, increased Leydig cell numbers	Enlarged seminal vesicle	High sperm counts	Fertile	(46)
*c-ros ^−/−^*	Transgenes	Head of epididymis			Low columnar epithelial cells in the epididymal head, reduced epididymal head size and weight		Infertile	(47–50)
*me^v^*/*me^v^*	Spontaneous mutations	Head of epididymis			Low columnar epithelial cells in the epididymal head, reduced epididymal head size and weight		Infertile	(51)
*c-neu*	Transgenes	Epididymis, seminal vesicle			Benign or malignant epididymal and seminal vesicle tumors		N/A	(52, 53)
*Ocln^−/−^*	Targeted mutations (homologous recombination)	Head of epididymis			Epididymis with enlarged head, degenerated tail, and granuloma-like tissue knot in the head-body junction	Low sperm counts, motility, and fertilizing ability	Infertile	(54)
*int-2*, *int-2* × *Wnt-1*	Transgenes	Prostate gland			Enlarged prostate gland (benign prostatic hyperplasia)		Infertile	(55, 56)
kgf	Transgenes	Accessory glands			Hyperplasia of vas deferens, seminal vesicle, and prostate gland		N/A	(57)
*Ttc29^mut/mut^*	Transgenes	Testis, spermatozoa				Low sperm motility, abnormal sperm morphology (bent flagella, abnormal flagella Ultrastructure), and low fertilizing ability	Subfertile	(58)
*azh/azh*	Spontaneous mutations	Testis (germ cells)		Spermatids with abnormal head shapes		Abnormal sperm morphology (club/crescent-shaped head, detached head, and coiled tail)	Possibly infertile	(59)
*Y^del^*	Spontaneous mutations	Testis (germ cells)		Degenerated seminiferous tubules, large testis		Abnormal sperm morphology (flat acrosome with less acrosomal enzyme, cytoplasmic droplet), poor capacitation ability, irregular movement, and low fertilizing ability	Subfertile	(60–62)
PRR^−/−^	Targeted mutations (knockout)	Testis (germ cells)				Acrosome with less acrosomal enzyme, low fertilizing ability	Subfertile	(63)
*Csnk2a2^−/−^*	Targeted mutations (homologous recombination)	Testis (germ cells)		Increased cell apoptosis, decreased germ cell numbers, elongating spermatids with deformed nuclei and acrosomes		Low sperm counts, abnormal sperm morphology (round head, bent flagella), and irregular movement	Infertile	(64)
*Actl7* ^amut/mut^	Targeted mutations (knock-in)	Testis (germ cells)				Low sperm counts, abnormal sperm morphology (detached acrosome), low sperm motility, and abnormal sperm function (fail to fertilize oocyte)	Infertile	(65)
*Actl7a^−/−^*	Targeted mutations (knockout)	testis (germ cells)		Spermatids with deformed acrosomes		Abnormal sperm morphology (detached acrosome)	Infertile	(66)
*GM130^−/−^*	Targeted mutations (knockout)	Testis (germ cells)		Spermatids with disorganized microtubules, round-shaped sperm heads in testis, decreased testicular size and weight		Low sperm counts, abnormal sperm morphology (round head without acrosome, loss of mitochondrial sheath) low sperm motility	Infertile	(67)
*Fsip1^−/−^*	Targeted mutations (knockout)	Testis (germ cells)		Elongated spermatids with abnormal head shapes and tails, few elongated spermatids		Low sperm counts, abnormal sperm morphology (misshaped heads without acrosome, short/absent tails), and low sperm motility	Infertile	(68)
*Ccnyl1^−/−^*	Targeted mutations (knockout)	Testis (germ cells)				Abnormal sperm morphology (bent head wrapped around the neck, thin annulus, microtubule breakage and extrusion) low sperm motility, and low fertilizing ability	Subfertile/infertile	(69)
*Lztfl1* KO	Targeted mutations (knockout)	Testis (germ cells)		Elongating spermatids with abnormal head shapes, few elongated spermatids		Low sperm counts, abnormal sperm morphology (misshaped heads, short tails, uneven tail thickness, and swollen tail tips), and slightly low sperm motility	Subfertile	(70)
*Ift74* KO	Targeted mutations (knockout)	Testis (germ cells)		Elongating spermatids with abnormal head shapes and tails, few elongated spermatids		Low sperm counts, abnormal sperm morphology (misshaped heads, short/absent tails), and low sperm motility	Infertile	(71)
*Ift81* KO	Targeted mutations (knockout)	Testis (germ cells)		Elongating spermatids with abnormal head shapes and tails, few elongated spermatids		Low sperm counts, abnormal sperm morphology (misshaped heads, short/absent tails), and low sperm motility	Infertile	(72)
*olt/olt*	Spontaneous mutations	Testis (germ cells)		Tail-less spermatids, decreased spermatid numbers, decreased testicular weight		Low sperm counts, abnormal sperm morphology (tail-less)	Infertile	(73)
*qk/qk*	Spontaneous mutations	Testis (germ cells)		Tail-less spermatids, decreased spermatid numbers, decreased testicular weight	Decreased seminal vesicle weight	Low sperm counts, abnormal sperm morphology (tail-less)	Infertile	(73)
Prml Gal	Transgenes	Germ cells				Abnormal sperm morphology (abnormal chromatin structure, tail-less, bent flagella), low sperm motility, and low fertilizing ability	Subfertile/infertile	(74)
*Pxt1^−/−^*	Targeted mutations (homologous recombination)	Testis (germ cells)				Slightly low sperm counts, abnormal sperm morphology (increased DNA strand breaks)	Fertile	(75)
BKO	Targeted mutations (knockout)	-				Spermatozoa with high ROS levels	Fertile/subfertile	(76)
*Cyrn^−/−^*	Targeted mutations (homologous recombination)	Testis (germ cells)				Abnormal sperm function (fail to bind and penetrate ZP)	Infertile	(77)
*fertilin β^−/−^*	Targeted mutations (homologous recombination)	Testis (germ cells)				Abnormal sperm function (defective migration to the oviduct, fail to bind to ZP and oocyte plasma membrane)	Infertile	(78)

M, Month; wk, week; FSH, Follicle stimulating hormone; LH, Luteinizing hormone; E, Estrogen; T, Testosterone; and ZP, Zona pellucida.

## The reproductive phenotypes in the level of the brain

**Figure 1:**
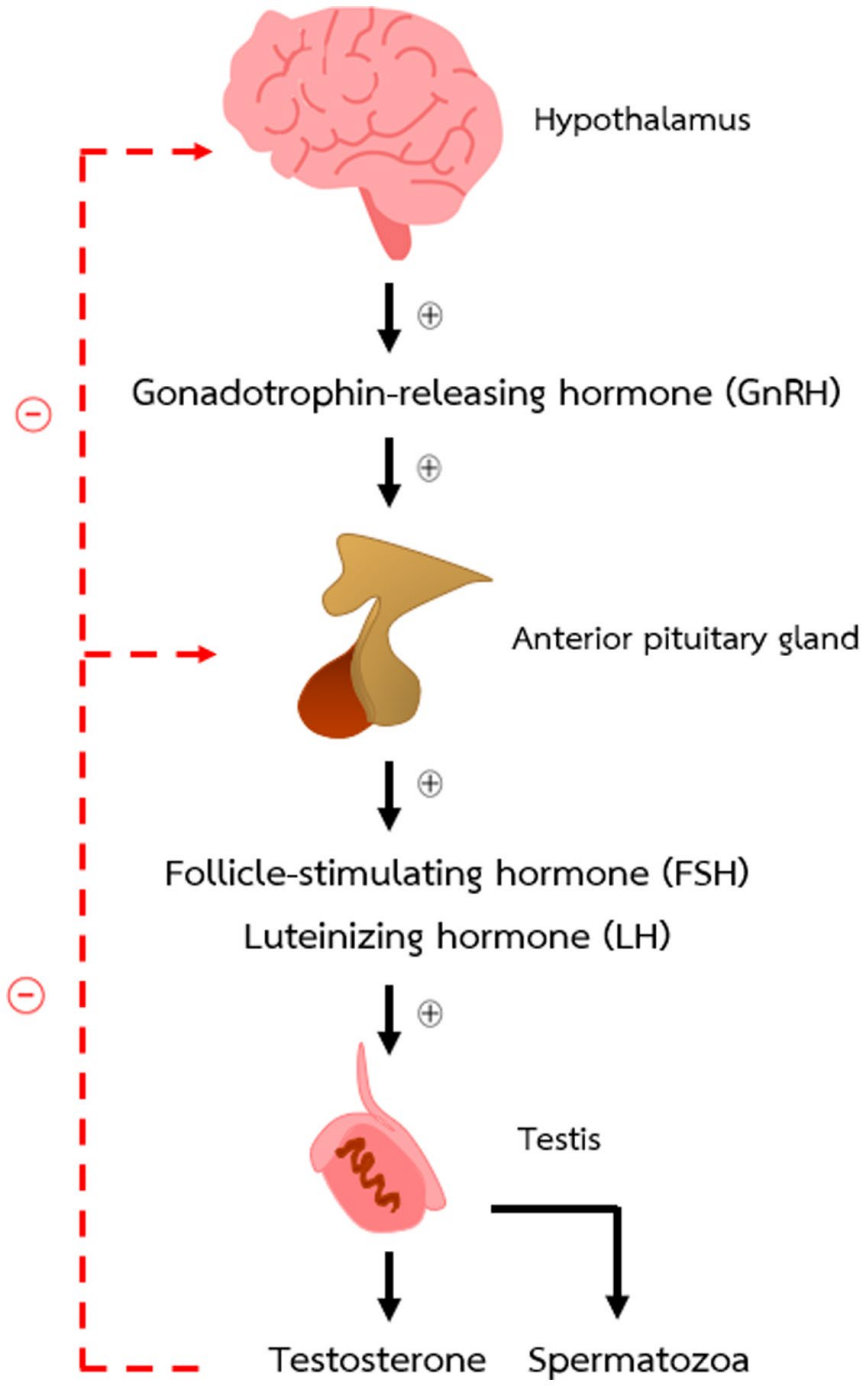
The hypothalamic–pituitary–gonadal axis (HPG axis) in male mice.

Two structures of the brain that have a pivotal role in controlling male reproductive functions are the hypothalamus and the anterior pituitary gland. Gonadotrophin-releasing hormone (GnRH) produced by the hypothalamus can activate or inhibit the production and secretion of follicle-stimulating hormone (FSH) and luteinizing hormone (LH) from the anterior pituitary gland. Both gonadotrophic hormones act on the gonads to stimulate sex hormone production and development of germ cells. The relationship between these two parts of the brain and the gonads is known as the hypothalamic–pituitary-gonadal (HPG) axis and shown in Figure 1.

There are some genetically altered mouse strains reported thus far having abnormalities in the hypothalamus and/or anterior pituitary gland and therefore resulting in the altered reproductive phenotypes. Mice homozygous for a null mutation in the gene encoding the major macrophage growth factor, colony-stimulating factor-1 (*Csfm^op^*/*Csfm^op^* mice) have impaired hypothalamic–pituitary feedback response to sex steroid hormones, have low serum LH and, in female mice, have no LH surge (9). Reproductive defects in *Csfm^op^*/*Csfm^op^* mice which will be described in detail later are primarily resulted from impaired microglia (specialized macrophages in the brain) functions that play a role in establishing functionality to the HPG axis, and are also highly related to dysfunction in the GABAergic system (10, 11).

The primary defect in the hypothalamus adversely influencing reproductive traits has also been reported in Gpr54 and Kiss1 knockout mice. These two genetically altered mice of both sexes show varying degrees of hypogonadotrophic hypogonadism, low levels of FSH and LH secretions, a complete absence of LH pulses, and thus infertility (14). The GnRH neurons in the hypothalamus of these mice are anatomically and numerically normal and synthesize GnRH regularly. However, the hypothalamus itself fails to secrete GnRH into the primary portal plexus to stimulate gonadotrophin release from the anterior pituitary because of depriving two key proteins: the G-protein coupled receptor GPR54 and kisspeptins, peptide ligands for this receptor encoded by the *Kiss1* gene (15). Failure of gonadotrophic hormone secretion in Kiss1 mutant mice can be treated by exogenous kisspeptin administration (79). On the other hand, such intervention was unable to significantly stimulate LH or FSH secretion in GPR54-null mice confirming that GPR54 is the principal receptor for kisspeptin action on the reproductive axis (80).

In contrast to Gpr54 and Kiss1 mutant mice, the GnRH neuron number within the hypothalamus of a GNR23 transgenic mouse strain which is generated by the integration of a transgene into chromosome 5 is reduced to approximately 12–34% of wild-type counterparts, resulting in reduced testicular weight and plasma FSH concentrations. Intriguingly, reproductive competency (litter number and size) in adult GNR23 male mice was normal and did not differ from that of wild-type mice (16). This indicates that just approximately 12% of the GnRH neuron population is sufficient to maintain reproductive performance in male mice. Differently, at this level of GnRH neurons, adult (>60 day-old) GNR23 female mice exhibit abnormal hormonal profile and subfertility such as reduced basal and mean plasma LH levels, absent LH surge, and no ovulation (81). Ovulation failure, along with other reproductive problems, found in GNR23 female mice is a consequence of insufficient numbers of GnRH neurons to generate GnRH that, in turn, evokes the LH surge and ovulation. Together, these observations demonstrate that female mice, compared with males, require more GnRH neurons to drive and sustain normal reproductive function.

Mice deficient for Cyclin-dependent kinase 4 (*Cdk4^−/−^*), which were intentionally generated to understand the specific role of Cdk4 in regulating the cell cycle *in vivo*, display age dependent defects in pancreatic beta cell and reproductive functions. By 8 months, *Cdk4*-deficient mice exhibit severe beta cell hypotrophy, reduced insulin production, and elevated serum glucose levels (17). Defective glucose metabolism, which is a general characteristic of diabetes mellitus, negatively affects body weight gains and consequently normal function of all body systems including reproductive system and fertility. In the *Cdk4^−/−^* anterior pituitary, function of gonadotrophs (the cells that produce and release gonadotrophins) is disrupted. This may be responsible for a slight reduction in serum FSH levels (18) and the protraction of the estrus cycle in *Cdk4^−/−^* females (17).

A decrease in serum FSH levels because of pituitary disruption was also found in other genetically altered mice such as the follicle-stimulating hormone β-subunit knockout (*FSHβ^−/−^*), the activin type II receptor knockout (*ActRIIA^−/−^*), and mahogunin knockout (*md^nc^*) mice. Activins, a group of proteins, bind to the type II activin receptor at pituitary gonadotrophs and Sertoli cells in the testis and stimulate pituitary synthesis of FSH (82). Mahogunin, an important mediator of chromogenesis and neurodegeneration, is synthesized and widely exists in the reproductive tissues of C3H/HeJ strain wild-type male mice; it is responsible for regulating the activity of the HPG axis (21). The degree of FSH suppression and hence serum FSH levels are different between *FSHβ^−/−^* (no serum FSH) and *ActRIIA^−/−^* (33% of normal FSH levels) mice (19). However, both male mice have some common reproductive phenotypes such as normal fertility, complete spermatogenesis, and normal levels of serum LH and testosterone even though their testes were smaller, as a result of reduced sperm concentration, compared with those of wild-type animals (19, 20). In contrast, *mc^nd^* male mice which originally arose spontaneously in the C3H/HeJ strain have low levels of reproductive hormones (FSH, LH, estrogen, and testosterone) and are infertile. Sperm concentration and the percentage of actively progressively motile spermatozoa are decreased. The reproductive change in mahogunin deficient mice is highly attributed to spongiform degeneration of the pituitary gland (21).

## The reproductive phenotypes in the level of the gonads

Testis or Testicle is the male reproductive gland in all mammalian animals including mice that is associated with sperm and sex steroid hormone production and therefore male reproductive phenotypes. It comprises two internal structures: (1) convoluted seminiferous tubules which are responsible for sperm production and (2) the interstitial tissue where blood vessels, lymphatics, and Leydig cells are located and sex steroid hormones are produced. There are a number of literatures describing the observable reproductive characteristics of male mice in the level of the gonads. In this review, changes in testicular phenotype of the genetically altered mice are classified into negative (decreases in size and/or function) and positive (increases in size and/or function) alterations.

### Negative phenotypic alterations of the testis

In almost all cases, low testicular weight comes together with testicular atrophy. These anomalies were reported in many genetically altered mice, with various mechanisms. However, this is except for GNR23 mice and *Sdc1^+/−^* mice with reduced expression of a protein Syndecan-1 that participates in human pregnancy associated pathologies. The testes of both genetically altered mice along with the epididymides of *Sdc1^+/−^* males were low in weight but normal in microscopic anatomy (16, 27). Consequently, the sperm quality and perhaps fertility existed in the *Sdc1^+/−^* males were not compromised compared with wild-type animals (27).

Testicular weights in homozygous mutant males lacking the FSHβ gene (*FSHβ^−/−^*) or the ActRIIA gene (*ActRIIA^−/−^*) or both FSHβ and ActRIIA (FAR) decreased approximately 70% compared with wild-type or respective heterozygotes. This alteration was attributed to substantial declines (30–60%) in Sertoli cell and germ cell numbers in the testes which the degree of germ cell attrition increased in the later stages of spermatogenesis from spermatogonia to elongated spermatids (20, 83) and was more severe in FAR mice compared with the individual mutants (83). The defects in FSHβ-subunit gene and/or ActRIIA gene in genetically altered mice resulted in decreased serum FSH levels and thus diminished FSH action on both Sertoli cell proliferation and the capacity of Sertoli cells to nurture germ cells. Moreover, Kumar et al. (83) demonstrated that targeted disruption of ActRIIA gene adversely affects testicular function directly, independent of its actions via FSH homeostasis in the pituitary. Intriguingly, male mice lacking FSHβ and/or ActRIIA were still fertile and sired offspring, although the number of pups varied depending on genotype of mated females (19, 83).

Hox genes play a key role in the morphogenesis of several segmented and nonsegmented structures of the posterior region of the mouse body. Homozygous male mice bearing a disrupted Hoxa-10 gene (*Hoxa-10^−/−^*) are viable but, in the aspect of reproductive phenotypes, display high incidence of cryptorchid testes either unilaterally (60%) or bilaterally (40%) due to developmental abnormalities of the gubernaculum, a ligamentous cord that mediates testicular descent. In the latter case, no scrotal sac and reduced ano-genital distance could be observed (22). Histology of the testes revealed a marked reduction in the seminiferous tubule diameter, with reduced numbers and sloughing of spermatogonia and spermatocytes, absence of spermatids and mature sperm, and vacuolization of Sertoli cells (23). All of these defects were associated with unsuitable conditions including hyperthermia of the undescended testes and explain the infertility of male *Hoxa-10^−/−^* mutants. Interestingly, no macroscopic abnormalities were observed in the mutant females’ genital tract, although cystic uteri could be seen upon histological analysis and might account for the reduced fertility in some cases (22).

The CSF-1 nullizygous (*Csfm^op^*/*Csfm^op^*) mice have fertility defects as a consequence of severely depleted macrophage populations in many tissues including the hypothalamus and, in males, the testes. A serum testosterone concentration in the genetically altered mice is only 10% of the wild-type. This is caused by a loss of testicular macrophages that regulate the steroidogenic activity of the Leydig cells (12). Ultrastructure of the Leydig cells in *Csfm^op^*/*Csfm^op^* mice showed some striking abnormalities such as an unraveling of membranous whorls and/or dilation of the inter-membrane spaces, although the overall structure of the testis was normal (13).

Low testosterone blood levels were also noted in GATA-1 transgenic (ΔdblGATA) and Neuroplastin-deficient (*Nptn^−/−^*) male mice (25, 26). GATA-1 is a protein of the GATA family playing a crucial role in DNA binding and cell maturation and differentiation. In the male genital system, GATA-1 is expressed in the prostate gland and testis. Testicular histology of GATA-1 mice showed subtle changes in seminiferous tubules but displayed obvious alterations in interlobular compartment, which affect androgen production, namely increase in total Leydig cell numbers and decreases in Leydig cell size, blood vessels, and lymphatic space. GATA-1 males also presented other abnormal reproductive characteristics associated with reduced plasma testosterone levels including elevated LH concentrations, short anogenital distance, reduced seminal vesicle weight, and increased sperm head defects (25). In contrast, adult *Nptn^−/−^* males with decreased blood testosterone levels did not show any reproductive phenotypic abnormalities. The morphology and histology of the male reproductive tract as well as sperm characteristics of *Nptn^−/−^* mice were normal (26). The mechanism underlies behavioral immaturity (the complete absence of mating behavior) and therefore infertility of *Nptn^−/−^* males is a decrease in Plasma Membrane Ca^2+^ ATPase 1 (PMCA1) expression and a lack of PMCA-neuroplastin complexes in the mutant Leydig cells. Neuroplastin is a type I transmembrane protein belonging to the immunoglobulin (Ig) superfamily and, in the field of reproduction, is crucial for PMCAs expression, Ca^2+^ transport, and LH-mediated stimulation of testosterone production by Leydig cells.

Spermatogenesis in male *Atm*^*−*/−^ mice, that lack the protein kinase ataxia telangiectasia-mutated (ATM) regulating cell cycle checkpoints and apoptosis, is disrupted during early stages of premeiotic germ cells leading to chromosome fragmentation, germ cell degeneration, and eventually testicular atrophy. The ratio of the tubules having premeiotic spermatogonia decreased significantly compared with wild-type (*Atm^+/+^*) mice at 6 months of age (28). Furthermore, *Atm* disruption caused an immature nuclear architecture (telomere clusters) and heterochromatin distribution in Sertoli cells, detected by immunofluorescence staining (29). Male *Atm*^*−*/−^ mice are infertile due to spermatogenesis disruption during first meiosis.

Mice overexpressing the apoptosis-inhibitory Bcl-xL or Bcl-2 proteins in the male germinal cells display marked alterations in the late stages of spermatogenesis accompanied by sterility. Massive spermatogonia and a few spermatocytes were observed in the seminiferous tubules of the immature *Bcl-xL* or *Bcl-2* testes. In older transgenic mice, the testes reduced in size and weight, by 20–50% of wild-type mice. The seminiferous tubules, on histologic sections, showed a varying range of abnormalities including (1) the severe depletion or absence of developing and mature germ cells, with normal appearance and distribution of Sertoli and Leydig cells; (2) complete cellular depletion of the tubules; and (3) the presence of multinucleated giant cells in the tubules. The major histologic abnormalities could be observed from the age of 5 weeks onwards, but the severity of the lesions was unpredictable and was not related to the age of *bcl-xL* and *bcl-2* mice (30). Morphologic alterations of the testes in mice deficient for proapoptotic protein Bax (*Bax^−/−^* mice) were very similar to mice overexpressing the apoptosis-protecting Bcl-xL or Bcl-2 proteins, i.e., accumulation of the premeiotic germ cells and enlargement of the testes at an early age followed by degeneration of the accumulated cells and shrinkage of the testes at an advanced age (31). During late puberty, massive degeneration of Bax-deficient germ cells took place through an apoptosis-independent pathway that was triggered by overcrowding of the seminiferous epithelium (32).

In contrast to *bcl-xL* and *bcl-2* mice, a progressive age-dependent testes dysfunction is characterized in *Cdk4^−/−^*, ROSA41, *bcl-w*-deficient, *bcl-w* overexpressing, as well as c-FLIP_L_ mice overexpressing the long isoform of the apoptotic modulator c-FLIP (17, 33–36). The seminiferous tubules and epididymides of more mature (>4 months) Cdk4 mutant mice showed evidences of decreased spermatogenesis, increased germ cell apoptosis, and increased atrophy. The average testicular weight of 6-month-old *Cdk4^−/−^* mice was approximately one-tenth of the wild-types. Cdk4-deficient mice infertility is diabetes-related events (17).

Homozygous ROSA41 male mice, generated by the chance integration of a gene trap retroviral vector 134-nt upstream of *bcl-w* exon 3, exhibit sterility associated with progressive testicular degeneration. Spermatogenesis is arrested during late spermiogenesis in young adults. All elongating spermatids displayed extensive cytoplasmic vacuolation suggesting cellular degeneration, and no spermatozoa were observed in the testes and epididymides. Gradual depletion of all germ cell stages resulted in a Sertoli-cell-only phenotype by approximately 6 months of age. Thereafter, most Sertoli cells lost from the seminiferous tubules and the Leydig cell population decreased, causing seminiferous epithelium disorganization and the smaller testes and seminal vesicles compared with controls. Interestingly, plasma FSH, but not LH, levels in ROSA41 mice were almost twice as high as the controls and seemed steady over a lifetime (33). As analysis with a molecular technique demonstrates *Bcl-w* gene, an anti-apoptotic *Bcl-2* subfamily, inadvertently mutated in the ROSA41 line of mice, testicular defects in ROSA41 mice resemble those of *bcl-w*-deficient mice (34). The *bcl-w*-deficient testes reduced in size and lacked elongating spermatids and spermatozoa. However, compared with ROSA41 males, germ cell apoptosis in *bcl-w^−/−^* commenced later, and the number of Leydig cells increased in adult mice responding to disordered spermatogenesis. Moreover, the serum FSH, LH, and androgen concentrations of genetically altered mice fell within normal ranges, indicating that reproductive phenotype in *bcl-w*-deficient mice is not an endocrinological basis (34).

Testicular degeneration in mice overexpressing *bcl-w* somewhat differs from *bcl-w*-deficient and ROSA41 males in the aspect of onset and pattern of germ cell depletion, namely depletion of germ cells began earlier, starting at 1-week of age, and overpopulation of any type of germ cells (spermatogonia, spermatocytes, spermatids, and spermatozoa) was never observed during testicular development. Moreover, Sertoli cells of the *bcl-w* testes functioned properly. Overexpression of *Bcl*-*w* affecting cell cycle entry and/or cell cycle progression of spermatogonia was suspected to be a mechanism underlying the spermatogenic defects in the *bcl-w* transgenic mice (35).

Unilateral testicular atrophy is initially seen in c-FLIP_L_ mice, followed by bilateral atrophy when the males are older than 8 months. The regressed testes, under the microscope, contained atrophic seminiferous tubules with a range of abnormalities resembling those of *bcl-xL* or *bcl-2* mice. However, these abnormalities were noted in c-FLIP_L_ mice at 8 weeks of age (later than *bcl-xL* or *bcl-2* mice) and became more prominent in older mice, suggesting that aging induces a more severe phenotype (36).

Estrogen receptor (ER) and its ligand, estradiol, regulate fluid reabsorption in the head of the epididymis and are essential for fertility of male, in addition to female. Male genetically altered mice lacking ER gene known as the ER gene knockout (ERKO) mice display some abnormal reproductive phenotypes. In the level of the gonads, testicular weight was transiently increased during puberty as a result of testicular secretions accumulated in the lumen of the seminiferous tubule, rete testis, and efferent ductule, leading to decreased sperm concentration. By the age of 5–6 months, the testes became shrunken and atrophic caused by long-term accumulation and back-pressure of the luminal fluids (37). The accessory sex glands including the seminal vesicles and coagulating glands of ERKO males remained intact anyway (38). Rete testis dilation and swelling of the lumen of the tubule due to a fluid reabsorption failure were also observed in the leucine-rich G protein-coupled receptor 4 (*Lgr4*) hypomorphic mutant mouse lines (*Lgr4^Gt/Gt^* and *Lgr4^−/−^*). Such abnormalities are at least partially caused by a decreased ER expression in the efferent ducts of the homozygous mutant males (40, 41).

Vascular endothelial growth factor (VEGF)—a key regulator of endothelial growth and permeability—and VEGF receptors are expressed in vascular endothelial cells, certain spermatogenic cells (i.e., round spermatids), and the Leydig cells of male mouse reproductive organs. Overexpression of VEGF in transgenic male mice leads to structural alteration and impaired function of the reproductive system and ultimately infertility. Compared with histological analysis of the wild-type testes and epididymides, the seminiferous tubules of the 6-month-old VEGF males contained vacuoles and disorganized seminiferous epithelium. Some spermatogenic stages, i.e., spermatogonia, spermatocytes, and round spermatids could only be seen. Neither elongated spermatids nor spermatozoa were observed, due to spermatogenic arrest occurring at the elongation phase of the spermatids. Furthermore, the capillaries of the testis and epididymis increased in size, density (by approximately 60% of the wild-type), and permeability resulting in fibrin accumulation in tissues. Such capillary abnormalities may be, at least partly, responsible for the observed swelling of the rete testis and ductus epididymis. Interestingly, both Sertoli and Leydig cells remained normal, although the expression of VEGF receptor is also strongly upregulated in such cells. Taken together, the failure to produce offspring of the VEGF male mice results from VEGF acting on both vascular endothelial and spermatogenic cells in the male genital tract (42).

### Positive phenotypic alterations of the testis

The testicular phenotypes of mice have not only been described in light of decreased functionality, number, and/or volume but excessive growth and function of this reproductive gland have also been delineated. For example, male mice carrying both c-*myc* and *CDC37* transgenes displayed Leydig cell tumors and testicular hyperplasia at the age of as early as 10 months (43). The establishment of tumor in *CDC37*/c-*myc* double transgenic males is a result of both *CDC37* and *c-myc* oncogene expression, since expression of either *CDC37* or c-*myc* gene and nontransgenic wild-type males are typically free of proliferative disorders (84). *CDC37* is an essential gene required to support both normal and abnormal proliferation of cells in multiple tissues including the testis, and it enhances both the rate and extent of cell transformation by c-*myc* oncogene. Likewise, overexpression of cyclin D1 oncogene in cyclin D1 transgenic mice resulted in abnormal cell proliferation including mammary adenocarcinomas in females and a testicular sarcoma in males. However, on the contrary to c-*myc* and *CDC37* transgenes, hyperplastic changes in cyclin D1 mice have only been observed after a latency longer than 16 months which were the result of the transgene, not aging *per se* (44).

Cyclin Dependent Kinase 4 (*CDK4*) facilitates cell cycle progression by regulating retinoblastoma tumor suppressor (*RB*). The activity of kinase is restricted to the G1-S phase, which is controlled by CDK inhibitor p16^INK4A^. Mutations in *CDK4* and p16^INK4A^ that result in enhanced kinase activity and increased cell proliferation have been implicated in the genesis and progression of melanoma in humans (85). Homozygous *Cdk4*^R24C/R24C^ mice generated by introducing the R24C mutation in the *Cdk4* locus of mice by using knock-in technology developed spontaneous tumors of various etiology, starting from 8 to 10 months of their life span. The majority of these tumors were found in the liver, skin, brain including pituitary, and mammary gland. In homozygous male mice, Leydig cell tumor was observed in almost one-fourth (23.8%) of the population and approximately 5% of homozygous males developed adenoma of the seminal vesicles. It is interesting that heterozygous *Cdk4*^*+*/R24C^ mice also developed spontaneous tumors in multiple organs, albeit at a lower frequency and incidence (45). The reproductive phenotypes of *Cdk4*^R24C^ knock-in mice are paradoxical to *Cdk4*-deficient (*Cdk4^−/−^*) mice, which display hypoproliferation of the gonads (17).

Mice possessing a null mutation at the *Prl3c1* locus, a member of the prolactin gene family, called *Prl3c1*-null mice exhibited an overgrowth of male reproductive tract. At the 2–5 months, gross weight of the testis and testis-to-body weight ratio were significantly greater in *Prl3c1*-null mice than wild-type counterparts. The enlarged testes in knockout mice were associated with, when observed microscopically, an expansion of interstitial compartments and proliferation of the Leydig cells; the latter was responsible for an approximately sevenfold increase in serum testosterone levels compared to those of the wild-type males (46). Levels of serum LH—a reproductive hormonal product of the anterior pituitary that drives the Leydig cells to produce testosterone—were also significantly elevated in mutant animals. The *Prl3c1*-null male reproductive phenotype demonstrates the impact of *Prl3c1* gene in establishing a homeostatic setpoint control system programming testicular growth and function in the mouse. It is noteworthy that, despite an altered reproductive tract phenotype, *Prl3c1*-null and wild-type males exhibited similar fertility. The pregnancy rate of wild-type females was 100 and 79% as mated with wild-type and *Prl3c1*-null male mice, respectively (46).

## The reproductive phenotypes in the level of the epididymides, accessory sex glands, and external genitalia

### Epididymal phenotype

The epididymis is a single, narrow, tightly-coiled tube that connects the testis to the vas deferens in the male reproductive system. It consists of three main regions, i.e., head (caput), body (corpus), and tail (cauda) which involve in sperm maturation processes necessary for fertility. In rodents, epididymal transit takes 10–13 days (86). The epididymal phenotype in mice has been focused on just some specific regions or the entire tube, with hypofunction or hyperfunction.

Both the *c-ros* protooncogene, encoding the transmembrane tyrosine kinase Ros, and the SHP-1 gene, encoding the SH2 domain protein tyrosine phosphatase SHP-1, are co-expressed at the initial segment of the epididymal head and play a vital role in controlling appropriate development of epithelial cells in the epididymis particularly regionalization and terminal differentiation. Therefore, a phenotypic abnormality of the reproductive tract in homozygous c-ros knockout mice (*c-ros ^−/−^*) and SHP-1 mutant viable motheaten mice (*me^v^*/*me^v^*) was found to be nearly identical (47, 51). Upon external examination, the epididymal head in the mutant animals reduced in size and weight when compared with the caput of control littermates (48). Histological analysis revealed distinct low columnar epithelial cells instead of tall columnar cells with long microvilli found in control animals (49, 51). These epididymal defects interfere with its normal function in resorption and secretion of testicular fluid and epididymal-specific proteins (87) and consequently adversely affect sperm maturation and fertilizing ability *in vivo* (50, 51). Intriguingly, heterozygous male mice are indistinguishable from the wild-type and remain fertile (47); also, the fertility of *c-ros ^−/−^* females is not affected (49). Developmental defect in the epididymis of *c-ros ^−/−^* and *me^v^*/*me^v^* mice was similar to that of *Lgr4* mutant males, despite with different mechanisms, in the aspect of the short and distended caput epididymis with undeveloped initial segment and the decreased epididymal weight (40, 41). In addition to such phenotypes, a large cystic epididymis found in *Lgr4* mutant mice was surrounded by a thick condensation of mesenchymal cells and rupture of swollen epididymal ducts was sometimes observed, leading to massive immune cell infiltration into the lumen viewed on microscopes. Infertility of *Lgr4* mutant males was owing to the combination of a developmental defect of the epididymal head and the rupture of the epithelium resulting in decreased motility and abnormal morphology of spermatozoa. The expression level of a key molecule ER1 that regulates water reabsorption, in the epithelial cells of the caput epididymides was much lower in *Lgr4^Gt/Gt^* males than in wild-type animals (40).

On the contrary to undeveloped epididymides of mutant mice mentioned above, the testes and epididymides of the Cdk4 deficient (*Cdk4^−/−^*) males are fully developed and functioned well. This was indicated through abundant mature sperm found at 2–3-month-old. However, as age progresses, the histology of the seminiferous tubules and epididymides of many *Cdk4^−/−^* mice showed evidence of increased atrophy with large abnormal apoptotic bodies and very few or no normal spermatozoa (17). Reproductive dysfunction and sterility in the older *Cdk4^−/−^* mice are associated with the deficiency of pancreatic β-cell development and a diabetic-like phenotype, which the severity increases with age.

Overexpression of the *c-neu* tyrosine kinase receptor oncogene in tissue of genetically altered mice results in diverse effects. Regarding the genital tract, the *c-neu* expression in the epididymides and accessory glands (seminal vesicles) led to the phenotypes of benign, bilateral epithelial hypertrophy and hyperplasia (52), or multifocal neoplasia (53). In contrast, expression of the activated *c-neu* in breast tissue induced mammary adenocarcinomas in some males and all females independently from pregnancy (53, 88). These data indicate that the Neu oncogene can induce tumors in all the reproductive tissues where it is overexpressed and that types of tumors (benign vs. malignant) are dependent on tissue context and a transgenic mouse lineage. The effect of *c-neu* expression on male mice fertility has not been reported thus far.

The epididymides of genetically altered mice with overexpression of VEGF, a major regulator of blood vessel growth and permeability, are abnormal in addition to the testes (42). In the 6-month-old VEGF males, the enlarged and swollen epididymides were noticed via gross observation. Histologically, the ductus epididymides especially in the head and body were dilated, containing areas of epithelial hyperproliferation. The epithelium of the epididymal head reduced in height, identical to that found normally in the cauda region. Blood vessels surrounding the epididymal duct increased in size and number. The permeability of subepithelial capillaries increased resulting in fibrin deposition in tissues. The disrupted function of male reproductive organs and consequently infertility are due to VEGF action on both endothelial and nonendothelial cells of the male genital tract.

Occludin (OCLN) is an epithelial tight junction protein enriched in the proximal regions of epididymis. *Ocln* knockout (*Ocln^−/−^*) male mice are infertile because of impaired epididymal function, which adversely affected the number, motility, and *in vitro* and *in vivo* fertilizing ability of spermatozoa (54). Based on integrative omics analysis, gene clusters enriched in acid secretion and fatty acid metabolism in the mutant epididymis, especially the enzymes related to the unsaturated arachidonic acid pathway were downregulated. Moreover, starting from prepubertal age before sperm entry, the number of proton-pump V-ATPase-expression clear cells—a key player of luminal acidification in the epididymis—declined markedly resulting in increased pH in the epididymal fluid and disrupted sperm maturation (54, 89). The microscopic examination of the mutant epididymis revealed the enlarged caput, the degenerated corda, and granuloma-like structure in the caput-corpus junction, which was caused by massive accumulation of sperm cells in the interstitial area. Intriguingly, infertility of *Ocln^−/−^* male mice could be counteracted by intracytoplasmic sperm injection (ICSI) bypassing normal fertilization process.

### Male accessory gland and external genitalia phenotypes

**Figure 2:**
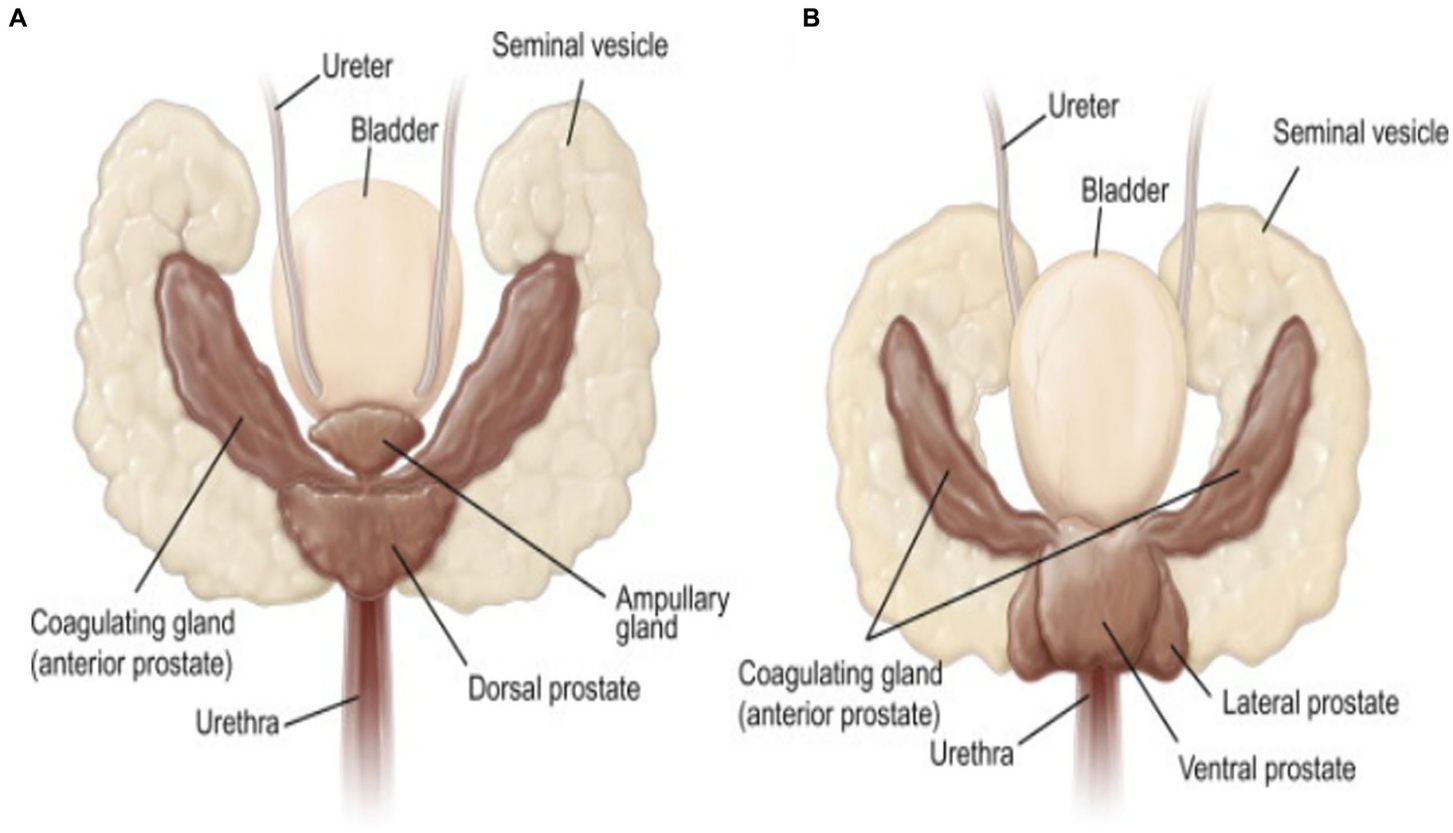
Dorsal **(A)** and ventral **(B)** views of the seminal vesicles, coagulating glands, and prostate gland (called accessory sex glands) in male mice [reproduced from ref. (90)].

During ejaculation, spermatozoa stored in the cauda epididymides enter the ejaculatory ducts via the vas deferens and eventually go out of the body through the external genitalia, i.e., the urethra and penis. As spermatozoa pass by the prostatic sinus, fluids rich in nutrients and energetic substrates secreted from the accessory sex glands are added. These additions activate sperm movement and help maintain sperm motility. Similar to other mammalian species, the seminal vesicles, prostate gland, and coagulating glands are accessory sex glands found in rodents (Figure 2) (90). The striking phenotypes of the accessory glands and the external male genitalia have been discovered in some of genetically altered mice and will be explained in the following sections.

The role of *Hox* genes in development of the male accessory sex organs was examined in knockout mice deficient for *Hoxa-10* function (*Hoxa-10^−/−^*) (24). The genes are important for patterning segmented and non-segmented body structures during embryonic development (91). *Hoxa-10^−/−^* male mice exhibited abnormal contour of the seminal vesicles and coagulating glands (also called the anterior prostate). The seminal vesicles of adult homozygous mutants, despite normal in length and diameter, were incompletely formed and lacked the complex clefting. The coagulating glands decreased in size and ductal branching. Histologic examination, however, revealed normal differentiation of the mutant seminal vesicle stroma and epithelium and normal appearance of the secretory product within the lumen, suggesting normal function of the glands. These data indicate the requirement of *Hoxa-10* for normal morphological development of the accessory sex organs. Subfertility/infertility of *Hoxa-10* deficient males reported by Satokata et al. (23) seems not associated with abnormal accessory sex gland morphology, but rather is related to cryptorchidism.

Homozygous ROSA41 male mice which are deficient in *Bclw* gene and genetically altered mice with mutations in Gpr54 or Kiss1 exhibit sterility associated with progressive testicular degeneration. In addition to the testes, the accessory glands and/or penis in homozygous mutants reduced in size and weight compared with controls as a secondary result of, for ROSA41 mice, a loss in paracrine signals from depleted Sertoli cells (33) and of, for the Gpr54 and Kiss1 mutant mice, a disorder of the hypothalamus to secrete GnRH (15). In ROSA41 mice, the seminal vesicle weight ranged from 100% of wild-type controls at 5-month-old to less than 10% at 14 months (33). Interestingly, heterozygous ROSA41 males were phenotypically indistinguishable from wild-type animals and the external genitalia of homozygous mutants retained normal phenotypes. The latter is, however, not the Gpr54 and Kiss1 adult males’ characteristic, which a small penis (called microphallus) and a reduced anogenital distance were found in all the mutant lines (15).

Instead of a regression, *Prl3c1*-knockout mice that deprive a *Prl3c1* gene significant for a homeostatic setpoint control system programming testicular growth and function displayed an age-dependent overgrowth of seminal vesicle glands, in addition to the testes. Compared with wild-types, a significant increase in seminal vesicle size was found in mutant mice as early as the age of 2 months. Further pathologic changes including hemorrhage and necrosis could be seen in some regions of the gland when *Prl3c1*-null males reached the age of 12 months. The excessive growth of the male reproductive organs, along with increased total sperm count, was highly related to elevated LH and testosterone levels in mutant mice serum (46).

Expression of the fibroblast growth factor (FGF)-related *int-2* transgene product in the prostate glands of *int-2* transgenic male mice results in a benign proliferative disturbance associated with male infertility. The transgenic prostates, according to microscopic examination, exhibited generalized epithelial hyperplasia and increased glandular secretions with the delayed onset and less severity found in *int-2* × *Wnt-1* bitransgenic males (55). Female transgenic animals that express high levels of *int-2* in the mammary gland also develop a pronounced enlargement of each gland during pregnancy. The enlarged prostate and mammary glands of affected animals have scarcely, however, become cancerous (55, 56). This is in contrast to keratinocyte growth factor (kgf), another member of the FGF family that frequently induces mammary and prostatic hyperplasia and mammary adenocarcinoma in kgf transgenic mice (57). The proliferation of the murine prostatic epithelium in *int-2* and kgf transgenic males is somewhat comparable to benign prostatic hyperplasia (BPH) found in human, suggesting that overexpression of *int-2* or kgf is capable of inducing epithelial proliferation and may play a role in the genesis of BPH. The identification of a role for *int-2* gene or a related gene in human is perhaps useful for BPH therapy.

## Sperm-related phenotypes in genetically altered mice

In general, mouse spermatozoa are made up of three segments: a head, midpiece, and tail. The head, with hook-like contour, consists of a nucleus of dense genetic material and acrosomal cap where various enzymes necessary for successful fertilization are stored. The midpiece contains mitochondria that supply the energy for the motility of spermatozoa. The tail, sometimes called the flagellum, is made of protein fibers including microtubules that give the sperm movement; additional energy is also generated here. A number of sperm characteristics, which are related to male fertility such as sperm quantity, motility, and morphology have been described in genetically altered mice.

### Sperm numbers

The number of spermatozoa harvested from the epididymides of mice rather varies depending at least partly on strain of mice. Most genetically altered mice that encounter fertility problems have lower sperm counts compared with control wild-type or heterozygote animals. For instance, homozygous *Csfm^op^*/*Csfm^op^* mice with substantially decreased testicular macrophages and serum testosterone had only 40% in sperm counts of the control (12); the transgenic males were also loss of libido and had low sperm viability (9). Fertility defects in male mutants indicate the close association of testicular macrophages with Leydig cells and the proposed function of these macrophages in the regulation of Leydig cell steroidogenesis.

Sperm counts in adult mice overexpressing *Bcl-w* and in mutant mice with ER-disrupted genes (ERKO) were about 3% (35) and 10% (38) of the control level, respectively. Disrupted spermatogenesis in *Bcl-w* transgenic mice is due to inhibitory effects of *Bcl*-*w* overexpression on germ cell cycle entry and/or cell cycle progression. The reduction of motility, concentration, and fertilizing ability of epididymal spermatozoa in ERKO mice could be either directly related to the loss of estrogen action on Sertoli cells and germ cells or indirectly caused by reduced fluid reabsorption in the efferent ducts resulting in seminiferous tubule dilation and subsequent degeneration (39).

Decreased epididymal sperm numbers (> 80% decrease) were also found in c-FLIP_L_ transgenic mice having atrophic testes (36). Such decrease is associated with the enhanced germ cell apoptosis and seminiferous tubule depletion as a result of c-FLIP_L_ overexpression. Interestingly, the sperm motility in c-FLIP mice was also impaired compared to age-matched control animals, and the motility reduction was more intense in c-FLIP_L_ mice showing atrophic testes than in those with normal-sized testes. The *in vivo* fertility in transgenic mice was, however, not affected.

### Sperm motility

The tetratricopeptide repeat domain 29 gene (*TTC29*) is preferentially expressed in the testis and plays an important role in cilia- and flagella-associated functions of the sperm cell. Spermatozoa of mice and men harboring *TTC29* mutations have severe asthenoteratospermia phenotypes. In the morphological analysis, spermatozoa with bent flagella were two times higher in mutant mice (*Ttc29^mut/mut^*) than the wild-type control. Furthermore, the lack of central-pair microtubules of the flagella was revealed according to transmission electron microscopy (TEM) observations (58). Abnormal flagellar ultrastructure in *Ttc29^mut/mut^* mouse spermatozoa is responsible for a significantly reduced sperm motility and decreased *in vivo* and *in vitro* fertility. Fortunately, the subfertility in *Ttc29*-mutated male mice could be overcome by ICSI.

Poor to zero sperm motility and infertility were also observed in the *Lgr4* null (*Lgr4^−/−^*) and hypomorphic (*Lgr4^Gt/Gt^*) mutant male mice. *Lgr4* is important for the regulation of ER1 – a key molecule that regulates water reabsorption in efferent ducts – expression in the genital tract. The mutant mice with a decreased expression of ER1 exhibit morphologic abnormalities in the seminiferous tubules, retia testes, and especially the epididymides (41) which result in decreased sperm motility and abnormal sperm morphology. Electron microscopic studies showed large cytoplasmic droplet and a hairpin structure at the sperm tails (40).

### Sperm morphology

Several genes are needed for the normal development of spermatozoa. According to the literatures, some mouse strains have certain types of morphologically abnormal spermatozoa as a result of genetic mutation or modification.

An autosomal recessive mutation in the murine *Hook1* gene is a cause of the abnormal spermatozoon head shape (azh) phenotype. Spermatozoa of the *azh/azh* mutant mouse displays an abnormal head morphology with club-shaped and crescent forms being the most common aberrations. Moreover, as *Hook1* is responsible for a close linkage between the microtubule, flagellum, and cellular structures, detached sperm heads and coiled sperm tails are frequently found in mutant *azh* mice. Histological analysis of the mice testes demonstrated a remarkable number of developing spermatids with abnormal head shapes (59).

Besides *azh* mice, male mice with a partial deletion of the Y chromosome (*Y^del^*) showed a substantial increase in the frequency of abnormal sperm heads compared to normal mice, and about 30% of abnormal spermatozoa were characterized by a flat acrosomal cap with a reduced concentration of proteolytic enzymes (60). Xian et al. (61) observed that the proportion of capacitated spermatozoa detected by chlortetracycline (CTC) assay was also significantly lower in *Y^del^* than in wild-type spermatozoa. The acrosomal abnormalities, poor capacitation state, as well as irregular movement of *Y^del^* spermatozoa negatively affected the success rate of *in vivo* (62) and *in vitro* (61) fertilization. The lack of a proteolytic enzyme, (pro)acrosin, in the acrosomal cap is also found in proacrosin-deficient male mice, that were generated by targeted disruption of the proline-rich region (PRR) of the proacrosin gene. Under normal *in vitro* fertilization (IVF) conditions, spermatozoa from PRR^−/−^ mice could still penetrate the zona pellucida and fertilize oocytes but with lower rates than those of wild-type males (63). In other words, PRR^−/−^ spermatozoa, at the same incubation period, showed a decreased IVF rate as compared with wild-type spermatozoa suggesting an important role of proacrosin in the acrosome reaction and thus fertilization success in mice. The morphology of PRR^−/−^ spermatozoa especially the head where the acrosome is located has not been assessed anyway.

In contrast, spermatozoa of infertile mutant mice lacking the casein kinase II α´ catalytic subunit (*Csnk2a2^−/−^*) and GM130, a cis-side localized Golgi matrix protein, (*GM130^−/−^*), and of homozygous actin-like 7A knock-in (*Actl7a^mut/mut^*) and knock-out (*Actl7a^−/−^*) mice still contained acrosomal contents, but the nuclear and/or acrosome morphology was deformed. By light and electron microscopy, the observable spermatogenic defect in the testes of *Actl7a* and *Csnk2a2* mutant mice began in round and elongating spermatids, respectively with abnormalities of the anterior head and became increasingly deformed with many acrosomes detaching from the nucleus as the germ cells proceed. In *Csnk2a2^−/−^* males, the mature sperm, besides misshapen (round) heads, had occasionally bent flagella with uncoordinated motility (64). Similarly, other than abnormal acrosome structure, reduced sperm motility was also a significant characteristic of *Actl7a*^*mut/*mut^, but not *Actl7a^−/−^*, mice (65, 66). Actin-like 7A is a member of the actin-related protein (ARP) family that participates in acrosome binding between the inner acrosome membrane and outer nuclear membrane of developing sperm cells; the mutations in this protein therefore result in the phenotype of peeling acrosomes. The same occurrence of acrosomal instability was described in other genetically altered mice such as *Actl7a^KI/KI^* (92), *Actl9^Mut/Mut^* (93), and mice lacking protein kinase *Hipk4* (94), actin-binding protein profilin 3 (*Pfn3*) (95), actin-related testis 1 (*Actrt1*) (96), actin-like 7B (*Actl7b*) (97), and *Actl7a* (98). The histology of the *GM130^−/−^* seminiferous tubules was grossly normal as compared with the control testes. However, all sperm cells in the testes and cauda epididymides from the mutants were round-headed, with complete absence of the acrosomes caused by failure of Golgi derived pro-acrosomic vesicles to fuse into a single large acrosomic granule. Additionally, the mitochondrial sheath in the mid-piece of the *GM130^−/−^* spermatozoa was not observed (67); this is probably responsible for the substantial declines in total and progressive motility of spermatozoa. Morphological abnormality in the head of *GM130^−/−^* spermatozoa was very similar to that of infertile *Gmap210*- (*Gmap210^flox/flox^*) (99), *Fsip1*- (*Fsip1^−/−^*) (68), and *Pdcl2*-knockout (*Pdcl2^−/−^*) males (100), which were recently created. Infertility in *Csnk2a2^−/−^*, *GM130^−/−^*, and *Actl7a^mut/mut^* male mice, once again excluding *Actl7a^−/−^*, was also related to oligospermia apart from abnormal sperm morphology and motility.

**Figure 3:**
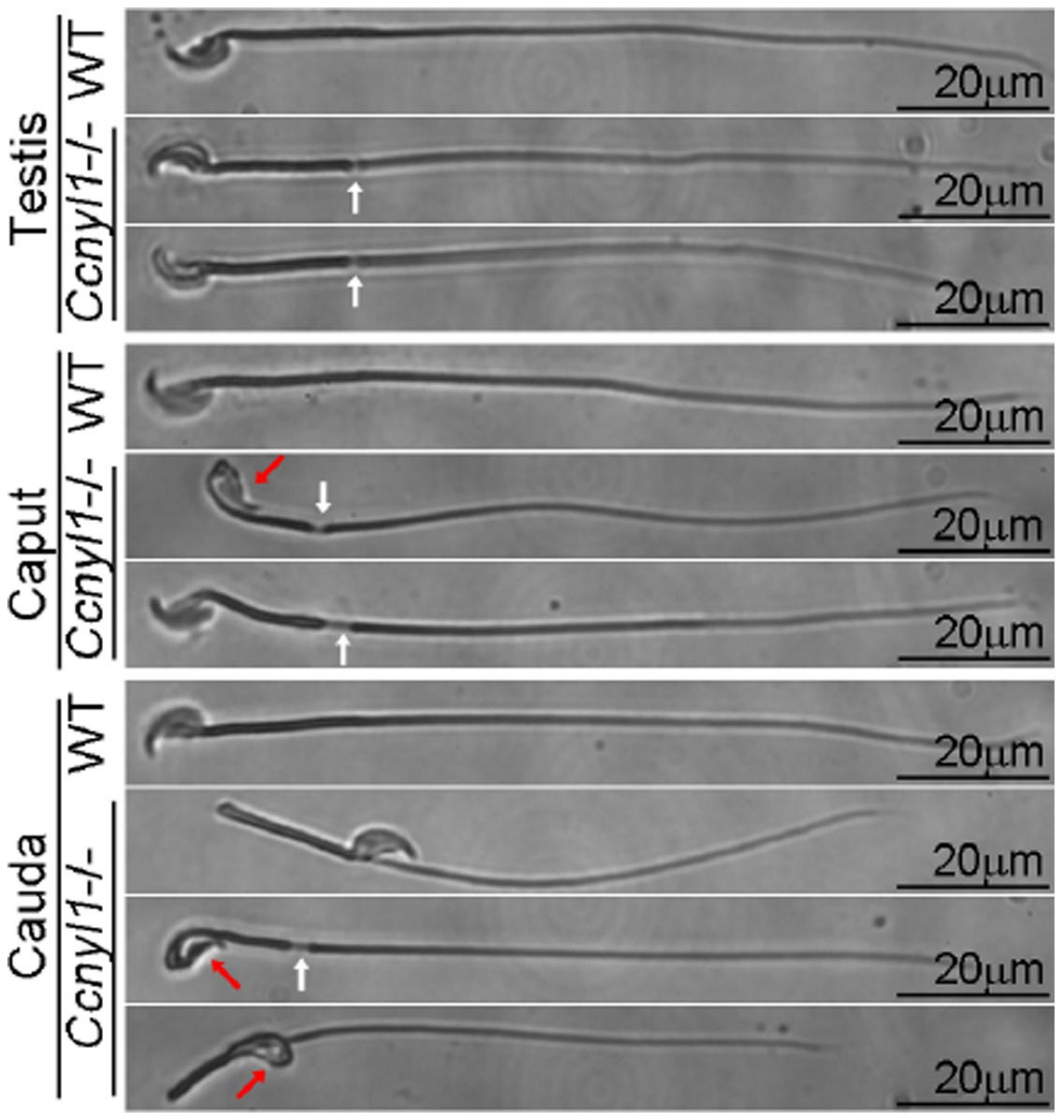
Phase contrast images of spermatozoa collected from the testis and epididymis (head and tail) of adult wild-type (WT) and *Ccnyl1^−/−^* mice. *Ccnyl1^−/−^* spermatozoa exhibit a thinning of annulus (white arrows) and a bent head wrapped around the neck (red arrows). Scale bar: 20 μm [reproduced from ref. (69)].

Cyclin Y-like 1 (*Ccnyl1*), a newly-identified member of the cyclin family, is highly expressed in the testis of mice and mainly localized on the plasma membrane of spermatocytes and spermatids. Almost all spermatozoa isolated from the caput and cauda epididymides of Ccnyl1 knockout (*Ccnyl1^−/−^*) mice displayed morphological defects. According to phase contrast and TEM images, many sperm heads were bent and wrapped around the neck. The body showed thinning and sharp bending of the annulus region with no mitochondrial sheath found at this locus (Figure 3). Microtubule breakage and extrusion were also observed (69). The structural defects of *Ccnyl1^−/−^* spermatozoa impaired the motility and velocity, that might prevent their propulsion and access to oocytes, and eventually led to impaired fertilization. Interestingly, female *Ccnyl1^−/−^* mice exhibited normal fertility. Spermatozoa of some mutant mouse strains displayed a quite similar phenotype to *Ccnyl1^−/−^* sperm cells in terms of a thinning annulus, a bent head, and a bent tail. However, additional defects of sperm morphology (101, 102) and of the caput epididymis functions (47, 103) were also detected and helped identify genotype of infertile mice.

In general, mouse spermatozoa have a very long tail to provide sperm motility, but this is except for those of male mice homozygous for the oligotriche (*olt/olt*) and quaking (*qk/qk*) gene mutations and of intraflagellar transport protein 74 (*Ift74*) and 81 (*Ift81*) and leucine zipper transcription factor-like 1 (*Lztfl1*) knockout mice. All the mutant mice had defective spermiogenesis, produced only tail-less or short-tailed spermatozoa, and hence were subfertile (70) or even sterile (71–73). Abnormal sperm tail development in the mutant males, despite with different mechanisms, was not a steroidogenic effect as Leydig cell function indicated through biochemical, physiologic, and/or microanatomic evidence remained intact (70–73). In male mice, IFT74, IFT81, and LZTFL1 (a binding partner of a major spermatogenesis regulator IFT27) are generally present in germ cells in the testis and are essential for sperm tail formation, probably through modulating the assembly and elongation of the sperm flagella. Besides sperm tail abnormalities, fertility problems in *Ift74*, *Ift81,* and *Lztfl1* knockout male mice were associated with low sperm count and decreased sperm motility (70–72). Interestingly, the *olt* and *qk* gene mutation action seemed specific for male fertility because female mice homozygous for either gene mutation were still fertile. Oligotriche and quaking mice could be differentiated visually through the thinning of the ventral coat and tremors, respectively.

### Sperm nucleus

In the haploid phase of mammal sperm formation, nuclear histone proteins originally found in somatic cells are substituted with small, arginine-rich, and highly positive charge nuclear protamine proteins. Such protamine features facilitate a tight binding between protamines and the nuclear sperm DNA and are essential for sperm head condensation, DNA stabilization, and protection of genetic material from damaging agents (104). Alterations of sperm nuclear components may therefore directly affect characteristics of spermatozoa as reported in Prml Gal transgenic mice that their sperm nuclei contain galline, the protamine of rooster spermatozoa. The sperm nucleus of Prml Gal mice was more vulnerable than that of wild-type mice. Chromatin heterogeneity and instability of the Prml Gal sperm nuclei led to the shorter timing of sperm chromatin decondensation. In addition, the motility of transgenic spermatozoa markedly decreased, and almost half of the sperm heads and tails were separated from each other after gentle pipetting (74). Altogether, during fertilization, spermatozoa of Prml Gal mice were not able to penetrate the zona pellucida as effectively as those of wild-type mice. The subfertility or infertility of Prml Gal males is directly related to amount of galline in the sperm nuclei and is all an effect of this protein (105). Compared with mouse protamines, galline has more positively charged arginine clusters and more affinity with DNA and, consequently, can disrupt disulfide bond formation which provides stability to mouse sperm chromatin structure.

The peroxisomal testis-specific 1 gene (*Pxt1*) is characterized as a gene with testis-specific expression and restricted to male germ cells during spermatogenesis in the mouse testis. The core function of *Pxt1* is related to the elimination of male germ cells with DNA strand breaks. Hence, it is unquestionable to find the characteristics of spermatozoa with increased DNA strand breaks in male mice with targeted disruption of the *Pxt1* gene (*Pxt1^−/−^*). Using Sperm Chromatin Structure Assay, proportion of epididymal spermatozoa with high DNA fragmentation index (DFI) was almost three times higher in *Pxt1* knockout as compared to control animals (75). Nonetheless, despite enhanced sperm DFI, mutants were still fertile and able to produce a normal number of viable offspring. Abortive apoptosis and/or oxidative stress, but not defective chromatin condensation, were suggested as cause(s) of increased sperm DFI in *Pxt1^−/−^* mice. Paradoxically, *Pxt1* overexpression induced cell death and resulted in male germ cell degeneration, leading to complete infertility of transgenic mice (106).

### Sperm oxidative stress

Mice heterozygous for the β-globin gene deletion (BKO mice) have been intentionally generated and used as a mouse model to study various aspects of β-thalassemia intermedia such as pathophysiology and novel therapeutic approaches. The classic signs and symptoms of β-thalassemia intermedia including, but not limited to, severe anemia, endocrine disorders, pulmonary hypertension, cardiac failure, and liver cirrhosis are direct and indirect effects of excessive reactive oxygen species (ROS) formation caused by the imbalanced α- or β-globin chains in the hemoglobin molecule and of excessive iron accumulation in the body caused by premature hemolysis and repeated blood transfusions. β-thalassemia patients with endocrine disorders exhibit slow growth, delay puberty, and diabetes mellitus, for example (107).

Excessive ROS levels have been reported in BKO mice mimicking the event seen in β-thalassemia patients (108) and, in terms of male reproductive function, can lead to sperm damage, poor sperm quality, and possibly infertility. Using the fluorescent dye, 2′,7′-dichlorodihydrofluorescein diacetate (H_2_DCF-DA) and flow cytometry to identify sperm oxidative stress, the number of viable spermatozoa with high levels of ROS in BKO mice was substantially greater than that of C57BL/6 wild-type mice (76). However, between wild-type and transgenic mice, no significant differences in the motility, morphology, and concentration of spermatozoa were detected. The indistinguishable *in vitro* sperm quality supported my colleagues’ preliminary study results that, without intraperitoneal iron administration, the levels of iron deposition in the body including the brain of BKO mice seemed not high enough to disrupt the normal function of the HPG axis that, as stated above, plays an important role in the regulation of spermatogenesis, sperm function, and fertility. Therefore, it can be suggested that iron supplementation is indispensable to BKO mice to mimic pathological events seen in β-thalassemia patients, which include male and perhaps female reproductive disorders.

### Sperm function

To achieve natural fertilization, the binding and fusion between spermatozoa and the oocyte plasma membrane are the indispensable events. The first interaction between a spermatozoon and an oocyte occurs at the zona pellucida, which a large number of sperm proteins are taken part in the interaction and lack of these proteins may affect fertilization success. Male mice with a homozygous disruption of *Cyrn* (*Cyrn^−/−^*) or fertilin β (*fertilin β^−/−^*) gene have normal sperm production and normal sexual behavior but are infertile. The infertility of *Cyrn^−/−^* males is due to the failure of mutant spermatozoa to bind and penetrate the zona pellucida which is a role of cyritestin, a membrane-anchored sperm protein encoded by the *Cyrn* gene on mouse chromosome 8 localized in the acrosomal region and participated in cell–cell adhesion. However, after removal of the zona pellucida, the rates of sperm-oocyte membrane fusion and embryonic development were comparable between *Cyrn^−/−^* and wild-type spermatozoa (77), suggesting that cyritestin is crucial in the fertilization process at the level of the sperm-zona pellucida interaction but is not required for sperm-oocyte plasma membrane binding and early embryonic development. This is difference from *fertilin β^−/−^* males which infertility is the combined effect of multiple sperm dysfunction including sperm migration from the uterus into the oviduct, sperm-zona pellucida binding, and sperm-oocyte plasma membrane adhesion and fusion (78). Fertilin belongs to the protein family involved in cell adhesion activity, the same as cyritestin does, but it is found on the plasma membrane instead of the acrosome of mammalian spermatozoa.

## The challenge of practicing gamete preservation in genetically altered male mice

The process of preserving male gametes in genetically altered mice with distinct reproductive phenotypes is probably different and more complicated than that of wild-type counterparts. Here are some important issues that must be taken into account when preserving and utilizing mutant male gametes.

### Age of male mice for gamete collection

Some genetically altered mouse strains such as *Cdk4^−/−^*, ROSA41, *Bcl-w^−/−^*, and c-FLIP_L_ experience age-related germ cell attrition and testicular dysfunction. Therefore, collection and preservation of male gametes must be performed on these mice prior to reaching such ages.

### Male gamete retrieval procedures

As some strains of genetically altered mice including *Bcl-xL, Bcl-2*, ROSA41, *Bcl-w^−/−^*, and VEGF have no or very few mature sperm cells, procedures for gamete collection and also stages of male gametes to be collected from these males must be adapted to collection of immature germ cells in the testis instead of collection of mature sperm in the epididymis conducted as usual.

### Selection of good-quality gametes before and after preservation

Decreases in sperm counts, motility, and/or viability are prominent sperm abnormalities occurred in some mutant mouse strains, as described above. Sperm selection, for instance, by swim up and density gradient centrifugation before and/or after preserving spermatozoa is therefore strongly recommended so as to eliminate dead/immotile cells and help improve success rate of preservation and, probably, fertility of preserved spermatozoa.

### The components of extenders for gamete preservation

Spermatozoa of some mutant males, such as BKO mice, containing high levels of ROS are especially vulnerable to preservation. Therefore, addition of exogenous antioxidant agents to sperm preservation media may be one of important steps to optimize sperm preservation protocol in this strain.

### Assisted reproductive techniques to produce offspring from the male gametes

Compared to those of wild-type animals, spermatozoa of several mutant mouse strains possess deviated characteristics (e.g., tail-less and round-headed acrosomeless) that hinder the process of natural fertilization between the oocyte and sperm cell. To produce offspring from such genetically altered males, assisted reproductive techniques, which in these circumstances is ICSI must be implemented to introduce a male gamete directly into an oocyte cytoplasm without requiring normal sperm tail function to propel the sperm toward the oocyte and acrosomal enzymes to digest the zona pellucida, which both are necessary for the natural fertilization process.

## Summary

A number of genetically altered mouse strains that have been models for human research exhibit distinct male reproductive phenotypes differing from wild-type animals. The altered reproductive characteristics involve decreases and increases in number, size, and/or function of just some or almost all organs of the male reproductive system and thus, to some extent, affect male fertility. Such reproductive alterations are either a direct effect of gene mutation such as cryptorchidism in *Hoxa-10* mutant mice or an indirect consequence of declined biological functions of other body systems induced by mutated genes such as diabetes-related infertility in *Cdk4*-deficient mice.

Comprehensive information about reproductive phenotypes of genetically altered male mice is a valuable tool for future biomedical research in terms of, for example, the selection of optimal animal models for the study in order to understand mechanisms, etiology, and clinical manifestations and therefore to discover effective prophylactic and therapeutic strategies for male reproductive system diseases and disorders. Furthermore, in view of mouse colony management, existing reproductive information provides helpful guidance for developing and optimizing male gamete preservation procedures for individual mouse strains. Points of concerned for achieving preservation and utilization of male gametes include, but are not confined to, age and conditions of male mice for gamete collection, the male gamete collection methods, selection and manipulation of good-quality gametes before and after preservation, the components of extenders for gamete preservation, as well as assisted reproductive techniques to produce offspring from the preserved gametes.

## Author contributions

KB: Conceptualization, Investigation, Writing – original draft, Writing – review & editing.
